# Pilot study of the early start of chemotherapy after resection of primary colorectal cancer with distant metastases (Pearl Star 01)

**DOI:** 10.1186/1477-7819-11-39

**Published:** 2013-02-07

**Authors:** Yoichiro Yoshida, Seiichiro Hoshino, Naoya Aisu, Masayasu Naito, Toru Miyake, Syu Tanimura, Yuichi Yamashita

**Affiliations:** 1 Department of Gastroenterological Surgery, Fukuoka University School of Medicine, 7-45-1 Nanakuma, Jonan-ku, Fukuoka, 814-0180, Japan

**Keywords:** Colorectal cancer, Chemotherapy, Surgery, XELOX, Early start

## Abstract

**Background:**

The start of chemotherapy usually requires a delay of about 4 weeks after surgical resection of colorectal cancer. However, there is no evidence for the required length of this delay interval. In addition, there is a chance that a patient may die because postoperative chemotherapy was not started soon enough and a metastatic tumor was able to develop rapidly. We therefore conducted a pilot study to determine the safety and feasibility of an early start of chemotherapy after the resection of colorectal cancer with distant metastases.

**Methods:**

Five patients were enrolled. They received XELOX therapy (130 mg/m2 of oxaliplatin on day 1 plus 1,000 mg/m2 of capecitabine twice daily on days 1 to 14) on the 7th postoperative day and XELOX + bevacizumab (7.5 mg/kg of bevacizumab on day 1) after the 2nd cycle of chemotherapy.

**Results:**

Five patients underwent open surgery. The procedures included right hemicolectomy in 1 patient, sigmoidectomy in 2 patients, high anterior resection in 1 patient, and Hartmann procedure in 1 patient. All patients started chemotherapy on postoperative day 7. The median number of cycles of chemotherapy was 11 (8 to 22). No postoperative complications were observed. The tumor reduction rate was 44.3% (32.0 to 66.6%). Progression-free survival was 10.3 months.

**Conclusions:**

An early start of chemotherapy after surgery is feasible and safe. These findings suggest possible changes in the start time of chemotherapy after surgery in the future. We have already started a new phase II trial to confirm the effects of the early start of chemotherapy after surgery.

**Trial registration:**

UMIN000004361.

## Background

The surgical resection of asymptomatic primary colorectal cancer (CRC) with unresectable synchronous metastases is controversial. There are no doubts that among patients with severe intestinal symptoms, resection is mandatory before starting systemic chemotherapy [1-3]. The palliative resection of the primary tumor has also been reported to improve the efficacy of systemic chemotherapy [4] and prolong the duration of chemotherapy [5]. A recent review article suggested that non-curative resection of asymptomatic colorectal primary tumors may prolong survival in patients with metastatic CRC [6]. However, another article concluded that initial chemotherapy should be started with resection of the primary tumor reserved for the small proportion of patients who develop major complications from the primary tumor because resection of an asymptomatic primary tumor provides only minimal palliative benefits [7].

The purpose of the surgical resection of primary tumors is the prevention of hemorrhage, perforation, and bowel obstruction. In many cases, it is not possible for patients to continue chemotherapy treatments because of complications, such as bleeding, perforation, and bowel obstruction, after chemotherapy is started without surgical resection of the primary tumor. Thus, it seems necessary to surgically remove a primary tumor in order to continue chemotherapy with few complications. However, surgical resection may delay the start of chemotherapy [8]. Generally, an interval of 4 weeks is considered necessary after an operation before beginning chemotherapy with treatments such as folinic acid, fluorouracil (5-FU), and oxaliplatin (FOLFOX); folinic acid, 5-FU, and irinotecan (FOLFIRI); and capecitabine and oxaliplatin (XELOX). However, there is no apparent evidence for this delay. A metastatic tumor can enlarge rapidly before the start of chemotherapy and possibly lead to patient death. Because the significance of the postoperative 4-week delay until the start of chemotherapy is not clear, we evaluated the feasibility and safety of an early start with chemotherapy in patients who had undergone colorectal surgery for CRC, and who had synchronous multiple distant metastases. This was a pilot study to exclude major problems with early administration of chemotherapy, and a phase II trial should be performed to confirm the safety and effects of the early start of chemotherapy after surgery.

## Methods

This study was designed as a pilot trial that was aimed at evaluating the feasibility of an early start of chemotherapy after surgery. After approval by our institutional review board, five consecutive patients who had primary CRC with synchronous distant metastases were enrolled. Further eligibility criteria included the following: (1) age 20 to 75 years; (2) Eastern Cooperative Oncology Group performance status of 0 or 1; (3) life expectancy of 3 months or more; (4) adequate hematological function (absolute leukocyte count 4,000 to 12,000 leukocytes/mm^3^; neutrophil count 2,000 neutrophils/mm^3^ or more, and platelets 100,000 platelets/mm^3^ or more), hepatic function (transaminases 2.5 times or less the upper limit of normal, and serum bilirubin 2.0 mg/dL or less) and renal function (serum creatinine below the upper limit of normal), and (5) ability to take oral medication. Patients were excluded if they had any of the following: brain metastases, history of other neoplasms (except cured non-melanoma skin carcinoma or cured carcinoma *in situ*), history of severe drug allergies, interstitial pneumonitis or pulmonary fibrosis, severe pleural effusion or ascites, active infection, diarrhea, or serious uncontrolled comorbidities or medical conditions.

**Figure 1:**
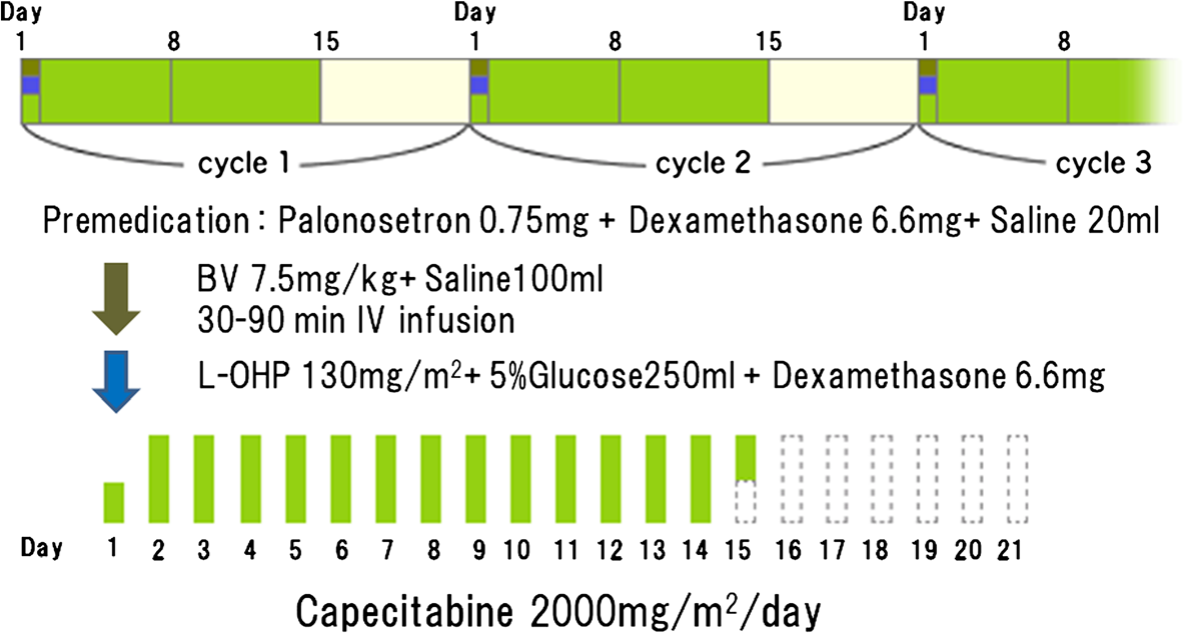
**XELOX plus bevacizumab regimen in the study.** Patients received XELOX therapy on the postoperative day 7 and XELOX + bevacizumab after the second cycle of chemotherapy.

Patients had received no prior chemotherapy. They received XELOX therapy (130 mg/m^2^ of oxaliplatin on day 1 plus 1,000 mg/m^2^ of capecitabine twice daily on days 1 to 14) on postoperative day 7 and XELOX + bevacizumab (7.5 mg/kg of bevacizumab and 130 mg/m^2^ of oxaliplatin on day 1 plus 1,000 mg/m^2^ of capecitabine twice daily on days 1 to 14, every 3 weeks) after the second cycle of chemotherapy (Figure 1), [9]. 

All patients underwent a physical examination, chest radiography, and computed tomographic scans of the abdomen, pelvis, and chest before starting treatment at baseline. All patients were included in the safety and efficacy analyses. The severity of the adverse effects was evaluated according to the National Cancer Institute Common Toxicity Criteria (NCI-CTC), version 4.0. Tumors were measured at 6- to 8-week intervals, and response was evaluated according to the response evaluation criteria for solid tumors (RECIST), version 1.1. The evaluation of response was based on radiologist-reported measurements. Complete and partial response required subsequent confirmation of response after an interval of at least 4 weeks.

The primary endpoint was postoperative morbidity within 30 postoperative days, and the secondary endpoints were response rate and progression-free survival (PFS). The following postoperative complications were predefined and prospectively measured: abdominal infection, anastomosis leakage, abdominal bleeding, and infection of the ileus or the wound. Other complications were recorded as appropriate. Hospital mortality was defined as postoperative death for any cause within 30 postoperative days, or death within the hospitalization period. The trial is registered with UMIN (Registration number, UMIN000004361). Written informed consent was obtained from all patients in advance.

## Results and discussion

This pilot study completed its accrual goal of five patients between December 2010 and April 2011. The minimum follow-up period was 12 months. Table 1 details the patient demographic and clinicopathological features. There were five men male patients with a median age of 67 years (range 50 to 72 years). All patients had tumors that were almost completely obstructive, with stenosis preventing the passage of the endoscope. Five patients underwent open surgery. A right hemicolectomy was performed in one patient, sigmoidectomy in two patients, a high anterior resection without a protective stoma in one patient, and a Hartmann procedure was performed in one patient. The median operating time was 130 minutes (range 75 to 225 minutes), and estimated blood loss was 60 mL (range 15 to 229 mL). The two patients who underwent sigmoidectomy had the longest operating times because of a history of previous surgery. A protocol for enhanced recovery after surgery (ERAS) was not applied in these five patients. All patients successfully started chemotherapy on postoperative day 7. No patient experienced any surgical complications within the first 7 postoperative days. The median number of cycles of chemotherapy was 11 (range 8 to 22 cycles). The median tumor shrinkage was 44.3% (range 32.0 to 66.6%). The response rate was 100%. The PFS ranged from 7.4 to 14.9 months with a median of 10.3 months (Table 2).

**Table 1:** Baseline characteristics

	**Patient 1**	**Patient 2**	**Patient 3**	**Patient 4**	**Patient 5**
Age	55	50	67	72	72
Gender	Male	Male	Male	Male	Male
Primary tumor	Sigmoid colon	Ascending colon	Rectum	Rectum	Sigmoid colon
Operation	Sigmoidectomy	Right colectomy	Hartmann	High anterior resection	Sigmoidectomy
Operating time, minutes	145	75	90	130	225
Bleeding, mL	60	15	130	60	290
Metastatic site	Liver	Liver	Liver	Liver	Liver
			Lung	Lung	

**Table 2:** Efficacy of chemotherapy

	**Patient 1**	**Patient 2**	**Patient 3**	**Patient 4**	**Patient 5**
Start, POD	7	7	7	7	7
Cycles	22	12	11	8	10
Response, %	66.6	44.3	32.0	33.3	46.9
PFS, months	14.9	12.6	8.1	7.4	10.3

POD, postoperative day; PFS, progression-free survival.

During the postoperative period, no patient developed surgical or medical complications due to the early start of chemotherapy. No in-hospital mortality occurred. NCI-CTC version 4.0 toxicity is described in Table 3. Two patients had grade 2 thrombocytopenia. Grade 1 hand-foot syndrome was observed in all patients. Grade 3 anorexia was observed in one patient. All adverse events were identified after the first seven postoperative days. No patient experienced other grade 3 or greater toxicities (Table 3). No patient experienced grade 2 or greater surgical complications or the chemotherapy toxicities in the first 60 days after colorectal surgery.

**Table 3:** Adverse events during chemotherapy

	**Patient 1**	**Patient 2**	**Patient 3**	**Patient 4**	**Patient 5**
Hematological					
Leukopenia	Grade1	-	-	-	Grade1
Thrombocytopenia	Grade2	-	-	-	Grade2
Non-hematological					
Hand foot syndrome	Grade1	Grade1	Grade1	Grade1	Grade1
Neuropathy	Grade1	Grade1	Grade1	-	Grade1
Nausea	Grade1	Grade1	-	-	-
Vomiting	Grade1	-	-	-	-
Anorexia	-	Grade1	Grade3		-
Hiccups	-	-	-	Grade1	-
Malaise	Grade1	-	-	-	-

The National Comprehensive Cancer Network currently recommends that patients with metastatic CRC undergo surgical intervention if they have a bowel obstruction, an impending obstruction, or metastases that are potentially resectable. Complications from the primary lesion are uncommon in these circumstances, and the removal of the lesion delays the initiation of systemic chemotherapy. Resection of CRC in patients with severe symptoms is mandatory before starting chemotherapy. In the past, some investigators have recommended routine resection of the primary tumor in order to prevent the need for urgent surgical procedures because of local complications [1,2]. Ruo reported that 30 (29%) of the 103 patients who were initially managed without bowel resection required a subsequent operation for the palliation of complications [10]. McCahill and Poultsides reported that only those cases with complete obstruction, perforation or massive bleeding (7 to 4%), require surgery of their primary tumor [11,12]. However, there is no report about an early start of chemotherapy after resection of primary CRC. Recently, some authors have suggested the elective resection of asymptomatic CRC in at least a subset of patients with less advanced stage IV disease [3,10]. Furthermore, Faron *et al*. reported the independent prognostic value on survival of primary tumor resection in patients with CRC and unresectable metastases [13]. Other authors have suggested deferring the resection of minimally symptomatic colorectal tumors because most of these patients succumb to progressive systemic disease instead of the complications related to the intact primary lesion [8,10,14]. We reported on a case involving an early chemotherapy start in a patient who had undergone right hemicolectomy for synchronous multiple liver metastases [14]. He survived for 22 months despite having huge liver metastases. We started this prospective study to confirm the feasibility of an early start of chemotherapy after surgery.

Surgeons are reluctant to prescribe 5-FU in the immediate postoperative period. This is primarily due to the belief that 5-FU will increase the anastomotic leakage rate. This can result in the need for reoperation, the creation of a colostomy, and the need for a future takedown, or even death. It is estimated that one out of three postoperative deaths after colonic surgery are due to a leaking anastomosis [15]. The dangers of postoperative 5-FU have been well documented. Several animal studies have documented a weaker anastomosis and the increased risk of anastomotic rupture when systemic 5-FU is given as a bolus immediately postoperatively [16,17]. Immediate intraperitoneal 5-FU also increases the risk of anastomotic dehiscence [18]. It has been documented that the continuous infusion of 5-FU allows for the use of greater daily dosages and appears safer than bolus administration of 5-FU [19]. Continuous infusion avoids the high peak serum levels of 5-FU that are seen with bolus dosing and may be effective for CRC without increasing the anastomotic leak rate. The oral fluoropyrimidine, UFT, and capecitabine, which have been developed to improve tolerability and patient convenience, have replaced the continuous infusion of 5-FU in many treatment regimens [20]. Oral fluoropyrimidine is a promising alternative to constant infusion of 5-FU, and pharmacokinetic studies have found that consecutive oral administration of UFT at 370 mg/m^2^/day given as tegafur, provided a steady-state concentration of 5-FU that was comparable to that achieved by a 5-day constant infusion at 250 mg/m^2^/day. In addition, injecting a bolus of 5-FU results in ultra-high concentrations followed by rapid disappearance [21,22]. Therefore, we chose the XELOX regimen for this study.

Currently, the precise timing for starting treatment with chemotherapeutic agents prior to and/or after surgery in order to avoid postoperative complications is not clear, but an interval of at least 4 weeks has been suggested. In most clinical trials, patients who had undergone an operation within 4 weeks were excluded. In Benoist’s study, the mean interval between diagnosis and the chemotherapy start was 44 days in the resection group versus 15 days in the chemotherapy group [8]. In our patients, although the operation was performed, the median interval between diagnosis and the chemotherapy start was 17 days. Resection of the primary tumor significantly increased the hospital stay and delayed the initiation of chemotherapy; however, there was no evidence to suggest that this delay was associated with reduced response rates leading to curative resection or reduced survival. However, there is a chance that patients may die if they are not able to start chemotherapy, because of the rapid postoperative progression of a metastatic tumor [23,24].

ERAS protocols aim at reducing the surgical stress response and optimizing recovery in order to reduce the length of hospital stays [25]. All elements in ERAS have separately been shown to improve patient outcome. Because ERAS was developed, an early start to chemotherapy after surgery was enabled. Early chemotherapy after surgery may therefore prevent tumor growth. Resection of colorectal tumors that involve severe stenosis and bleeding is the first treatment step in order to prevent complications related to these tumors. The European multicentre study, the colon cancer laparoscopic or open resection (COLOR) trial, aimed to assess the short-term and long-term outcomes after laparoscopic or open surgery for colon cancer [26,27]. Further early administration will be made possible by minimally invasive surgery for the treatment of advanced colon cancer. Early chemotherapy may extend the prognosis for patients undergoing laparoscopic surgery for colon cancer.

## Conclusions

To the best of our knowledge, this is the first pilot study to determine the feasibility of early chemotherapy after resection of primary CRC with distant metastases, albeit in a small cohort of well-selected patients. An early start to chemotherapy after surgery may be safe and may improve the prognosis of CRC patients with synchronous metastases. These findings suggest possible changes in the future to the time of starting chemotherapy after surgery. We have already started a new phase II trial to confirm the effects of the early start of chemotherapy after surgery.

## Abbreviations

CRC: Colorectal cancer; ERAS: Enhanced recovery after surgery; 5-FU: Fluorouracil; NCI-CTC: National cancer institute common toxicity criteria; PFS: Progression-free survival; POD: Postoperative day; RECIST: Response evaluation criteria for solid tumors.

## Competing interests

The authors declare that they have no competing interests.

## Authors’ contributions

YoYo conceived of the study, analyzed the data and drafted the manuscript; SH, ST and YuYa helped revise the manuscript critically for important intellectual content; NA, MN and TM helped collect data. All authors read and approved the final manuscript.
